# Research challenges in prehospital care: the need for a simulation-based prehospital research laboratory

**DOI:** 10.1186/s41077-019-0090-0

**Published:** 2019-02-13

**Authors:** Hanna Maurin Söderholm, Henrik Andersson, Magnus Andersson Hagiwara, Per Backlund, Johanna Bergman, Lars Lundberg, Bengt Arne Sjöqvist

**Affiliations:** 1 PreHospen - Centre for Prehospital Research, Faculty of Librarianship, Information, Education and IT, University of Borås, SE-501 90 Borås, Sweden; 2 PreHospen - Centre for Prehospital Research, Faculty of Caring Science, Work Life and Social Welfare, University of Borås, SE-501 90 Borås, Sweden; 3 School of Informatics, University of Skövde, Box 408, SE-541 28 Skövde, Sweden; 4 PICTA - Prehospital ICT Arena, Lindholmen Science Park AB, SE-402 78 Göteborg, Sweden; 5 Biomedical Signals and Systems, Department of Electrical Engineering, Chalmers University of Technology, SE-412 96 Gothenburg, Sweden

**Keywords:** Simulation, Laboratory, Prehospital

## Abstract

There is a need for improved research in the field of prehospital care. At the same time, there are many barriers in prehospital research due to the complex context, posing unique challenges for research, development, and evaluation. The present paper argues for the potential of simulation for prehospital research, e.g., through the development of an advanced simulation-based prehospital research laboratory. However, the prehospital context is different from other healthcare areas, which implies special requirements for the design of this type of laboratory, in terms of simulation *width* (including the entire prehospital work process) and *depth* (level of scenario detail). A set of features pertaining to simulation width, scenario depth, equipment, and personnel and competence are proposed. Close tailoring between these features and the prehospital research problems and context presents great potential to improve and further prehospital research.

## Introduction

This commentary article discusses the challenges of conducting research in the prehospital field and how prehospital simulation, currently underused for research purposes, has great potential to address these.

Prehospital care has changed rapidly during the last couple of decades. It has quickly transformed from a transport organization to an integrated part of the healthcare system [1]. This fast transition poses major challenges to the organizations [2]. Emergency medical systems (EMS) clinicians have to carry out more advanced patient assessment and interventions, compared to the old system which was more focused on a “load and go” strategy, with quick on-scene assessments and transports to the nearest emergency department (ED). In addition to more advanced care for critically ill or injured patients [3], EMS clinicians must also make decisions regarding the level of care, including alternatives for the patient such as stay at home with self-care advice [4], transport to primary care facility [5], transport to the nearest ED, or bypassing the ED for direct transport to specialist assessment or treatment center [6]. Hence, the broad range of clinical skills needed in order to be prepared for many different types of patients requires high-level clinical reasoning and decision making skills [7]. Prehospital care may also be conducted in difficult environments, in all kind of weather conditions, 24 h a day, and a long way from medical support [8, 9].

Despite increased research efforts in the prehospital area, there are still many unsolved research challenges [10]. When reviewing the evidence base for 11 important prehospital research topics (e.g., patient priorities and decision making, patient assessment and management, workforce safety and hazards, and information and performance measurement), the Department of Health, UK [11], found poor evidence in eight of the 11 topics, pointing towards a need for more and/or better research. Other studies, e.g., a Delphi study from the UK [10] and studies from Canada [12] and Australia [13] confirm these needs. Hence, further development of prehospital research is essential in order to reach an evidence level comparable to other parts of the healthcare sector.

Although these research gaps are partly due to prehospital care being a relatively young research area [2], they are more related to practical barriers to effective research in this context [14], e.g., trials involving acutely sick or injured patients who cannot consent to treatment or study enrolment. Furthermore, it is physically difficult for researchers to have control over the study, and low study protocol compliance among EMS clinicians has been reported [15]. Another problem is the lack of contextual knowledge among researchers engaging in prehospital research. Often, these have hospital-based backgrounds [12, 16] and thus limited knowledge regarding the prehospital research context. When designing realistic, high-validity simulations for research purposes, thorough knowledge about not only physical environments but also work practices, protocol, equipment, and culture is crucial. To do this, close collaboration between EMS professionals and researchers is necessary. As suggested by Leonard et al. [17], successful EMS partnerships in prehospital research are obtained by early involvement of EMS clinicians already during research planning and pilot studies. A simulation laboratory, such as the one proposed in this paper, could ideally work as a center of excellence or research hub for researcher-clinician partnerships.

The aim of this paper is to argue for the development and use of prehospital simulation laboratories and their potential to improve research in the field of prehospital care overall and strengthen the success rates for prehospital clinical studies.

## Simulation in prehospital research

Already in 2004, Gaba [18] discussed the future role of simulation as a research method, in particular for addressing central research areas such as organizational practices and human factors. He also highlighted the role of simulation in the development and evaluation of new equipment and methods in healthcare. The future is here, and with advances in healthcare simulation, there is now a potential for simulation in a number of research areas. To date, simulation-based research projects are relatively sparse in the prehospital research literature (excl. training). Furthermore, similar to the practices in how simulation traditionally is used for training purposes, they often focus on specific tasks or skills and/or are designed in a way that does not take the full complexity of the prehospital process and different contexts into account.

The area with the largest number of published prehospital research articles using simulation as a research tool is the assessment of different airway techniques with 59 published articles in the time period 1984–2012. Assessment of cardiac pulmonary resuscitation is another area, where simulation was frequently used with 35 published articles during the same time period [19]. Some studies have used simulation for technology evaluation. Brummer et al. [20] performed a randomized controlled simulation experiment to evaluate the effect of night vision goggles on advanced life support skills among paramedics. Teleconsultation from the scene was evaluated in a controlled simulation experiment by Skorning et al. [21]. Hagiwara et al. [22] used simulation in a randomized controlled trial to determine the effect of a computerized decision support system (CDSS) in compliance with guidelines and time spent on scene. The effects of video consultations in connection to advanced airway interventions were investigated in simulation-based experiments [23, 24]. Hagiwara et al. (forthcoming) used advanced simulation techniques to evaluate the effect of a CDSS in connection to the prehospital assessment of patients with stroke symptoms.

Simulation with advanced human patient simulators has been discussed as a suitable method for cognitive engineering and patient safety research, with emphasis on its advantage to provide a controlled laboratory environment without placing patients at risk [25]. There are some examples also from the prehospital field. Lammers et al. [26] used simulation to study patient safety risks in connection to prehospital assessment and treatment of pediatric patients. Simulation has also been used to study stress and the impact of stress on EMS clinicians in a couple of studies [27, 28]. In addition, cognitive processes among EMS clinicians have been investigated in simulation-based studies [9, 29, 30]. Manikins with temperature sensors have been used to determine the effect of wet clothing removal in prehospital care [31] and the impact of heat stress of chemical protective clothing in connection to prehospital resuscitation [32]. These simulation-based studies are good examples of what simulation can add to the prehospital research in the future.

## How can an advanced simulation laboratory contribute to prehospital research?

### Addressing the overall complexity of the research context

One of the biggest challenges when conducting prehospital research is the lack of control over several elements of the study. This includes difficulties with inclusion procedures where the responsibility falls under the EMS clinician, e.g., it is not feasible to add a research nurse or assistant to the existing ambulance team. Hence, the researcher has no direct access to or control over the process. There are many possible advantages to use simulation as a preparation before a clinical trial, e.g., pre-testing the study protocol procedures in realistic situations with the EMS clinicians involved in the data collection. It can also be used to improve training of the study protocol, increase participant enrolment, and decrease early protocol violations [33]. Hence, a simulation laboratory can provide means for improving the quality of prehospital clinical studies.

### Development and evaluation of new technology and methods

Development and evaluation of prehospital equipment and technology are examples of areas where simulation-based studies have great potential. Simulation has already been used in these areas, but the problem with some of the studies [22, 23] is the lack of contextualization in the simulation. The traditional prehospital simulation is often performed in a decontextualized environment where only one or at best a few phases of an ambulance mission are represented. If the goal of a study, for example, is to evaluate a decision support system, it is of great value to be able to study the effects in all phases of an ambulance mission. With a contextual simulation scenario where all phases in an ambulance mission are present and the simulation context induces a high degree of immersion to the participant, it is possible to closely study the effects of new technologies on, e.g., work process and teamwork. In simulations, it is also possible to evaluate technologies in a premature or early stage of development where necessary certificates for clinical use might be lacking. Another benefit for simulation evaluation is increased user involvement in the development process. There is a strong correlation between user-involvement and system success [34].

### Studies on patient safety

Simulation has been highlighted to be an effective approach to increase patient safety by training healthcare teams [35]. Simulation also has the potential to be an effective research method to closely study prehospital patient safety risks and to evaluate patient safety interventions. In simulation, it is possible to set up a scenario and run realistic experiments. In the analysis phase, the video recordings make it possible to study the patient safety issues thoroughly. Simulation can replace some data collected in a clinical setting, which may otherwise require many hundreds of hours of observations until a particular situation of interest occurs again naturally, as opposed to being provoked by the circumstances of a specific scenario. This is especially important in prehospital care, where it is difficult to predict the number and locations of the situations or patients of interest for a particular study. In a simulation laboratory, there are no issues with informed patient consent, especially when patient simulators are used (although standard consent would still be needed from anyone participating in a research activity, e.g., EMS clinicians or simulated patients). Obtaining informed consent from real patients [16], as well as from the EMS clinicians on duty, is a major obstacle in conducting prehospital research in real life. This is due to the characteristics of many ambulance patient groups, e.g., patients with stroke symptoms, cardiac arrest, or multiple trauma, from whom it is impossible to obtain consent from and thus are excluded from prehospital clinical research. Another possibility in line with this is to recreate an adverse event and be able to analyze the event closely.

### Organizational development and quality improvement

Another area for prehospital simulation laboratory related to research is organizational development. Prehospital organizations could use a simulation-based prehospital research laboratory in many creative ways. Before procurement of new equipment, the organization could use simulation to determine if the equipment is appropriately suited for the task. It could also help them in defining requirements and specifications of new equipment. When introducing new equipment, techniques, and methods, simulation can provide benefits such as developing and providing the user suitable process recommendations, e.g., for work processes or guidelines. Hence, the implementation of new medical guidelines is another area where simulation has an important future role [36].

## The features of a prehospital research laboratory

Due to the characteristics of the prehospital context, it is not optimal to use the same types of simulation-based facilities, such as the ones often used in hospitals or for training purposes where only one or two locations and/or rooms are represented, and many of the prehospital activities, tasks, and dimensions are excluded. We argue that to be able to conduct advanced prehospital simulation research, it is critical that simulations include the full context, phases, and details of the entire prehospital work process, e.g., different types of fidelity as proposed by Lioce et al. [37] and further discussed by Engström et al. [38], as well as advanced technology and equipment for high-quality data collection and analysis. A recent research interdisciplinary (serious games, information science, prehospital care) project (SAREK) [39] developed a simulation platform for prehospital training and research [40]. The simulation platform was created to support patient care provision in live role-playing scenarios using simulated patients (actors or patient simulators). The platform integrated physical spaces and rooms, components such as a real ambulance and EMS equipment, and equipment for visualization. It also included software support, such as wall projections, sounds, and images, for creating different types of scenario environments.

The goal of this system was to enable realistic prehospital simulation scenarios covering all phases and parts of an ambulance mission from dispatch to handover of the patient at the emergency department. The project aimed to increase both the *width* and *depth* of the simulation. The width means that all phases, as defined by Carter and Thompson [41] and Jensen et al. [42], included in a typical ambulance mission are represented. Lioce et al. [37] propose this as one important dimension when designing simulation scenarios or cases. The depth means that all activities in the phases are represented as realistically as possible. To achieve depth, several approaches were used such as the use of standard equipment, the use of technology for sound and environmental simulation, and an effort to reduce disturbing interaction with people not involved in the scenarios. This means, for example, that a scenario starts with an alarm call made through a simulated ambulance dispatch unit and that the participating ambulance team will receive information via radio calls in the car. Upon arrival on scene, the team will then have the same information that they usually have about what to expect (e.g., what type of scene/location, patient, initial symptoms, and potentially other circumstances). After a short transfer from the ambulance into the scene room, they have to make an initial assessment of the patient and the environment and use whatever cues there are to determine whether the place is safe or not before they initiate treatment. [38, 40, 43] This approach is different from the traditional way of doing prehospital simulation, where the team typically will get a lot of verbal information from a scenario instructor and then have to ask the instructor for environmental and/or additional information not possible to gather or interpret from the scenario design or training environment. The simulation platform was evaluated in two experiments and tested for immersion among the scenario participants [38, 43, 44].

When designing this type of facility, close collaboration is required between users of the simulation and the people designing and developing the simulation facility. [45] Here, users include both people *participating* in simulations and people who *manage* or *use* either the simulation facility or the participants. The use of participatory design approaches, in combination with establishing constructive long-term partnerships and arenas between researchers, designers, and EMS organizations are crucial. The authors’ own experience is that simulation as activity and place works as a collaborative hub or artifact that enables and facilitates common ground across professions and disciplines. Hence, the features of future prehospital research laboratories could be derived from a combination of the reviewed work and the SAREK project. Great emphasis and efforts should be placed on the simulation width and depth and the competence partnerships in order to reach a high level of contextualization. Next, we outline some ideas on how to achieve this.

### Simulation width

To be able to simulate all phases in an ambulance mission, the following features will be of importance:

- Fully equipped ambulance which also will function as a driving simulator
- Realistic equipment for dispatch and communication
- Rooms for on-scene simulation (house of outdoor settings)
- Rooms for the handover phase (emergency department, intensive care unit, etc.)
- Possibilities to carry out documentation in accordance with the standard practice, using realistic/real prehospital documentation systems

### Simulation depth

To be able to simulate realistic situations and to create an immersive environment, the following features will be of importance:

- Projection of image and sound during driving in the simulator
- Projection of image and sound to be able to create realistic and different on-scene scenarios
- Environmental noise and sounds including crowds, traffic, music, rain, thunder, fire/car alarms, discussion, or pets/animals
- A room for environmental simulation with possibilities to create different weather conditions such as rain, wind, and different temperatures
- Fully equipped emergency room
- Real ambulance equipment
- Facilities and equipment for makeup and dressing of actors
- Advanced simulation manikins of different ages

### Research equipment

Equipment for gathering, storing, and analysis of data

- Equipment for video and audio recording
- Programs for storing and analysis of video and sound material
- Movement tracking devices
- Eye tracking devices
- IT infrastructure which supports IT components used in prehospital care
- Equipment for monitoring simulation participants
- Facilities for interviews and debriefing
- Control room for running simulations

### Personnel and competence resources

The idea of a research laboratory is to not only provide facilities for research, but also *competence*—both when it comes to designing the facility, simulation activities and scenarios, and approaches to research studies. Hence, the personnel connected to the research laboratory are of great importance.

- Researcher with deep knowledge of the prehospital context
- Researcher with experience of simulation research
- Researcher with experience of information science
- Simulation expertise, e.g., simulation techniques and methods
- Computer technicians
- Simulation technology technicians

## Summary

Prehospital care differs a lot from hospital care in terms of physical environments, mobility, work processes, and range of patients. There are phases and tasks not present in hospital care such as driving and radio communication, and changing and unstable environments provide challenges to performing patient care. While there is a need for more research in the prehospital context, the context itself presents obstacles and difficulties to this, e.g., as lack of control and an insufficient EMS clinician involvement. Hence, we argue for the development of advanced simulation laboratories dedicated to the prehospital context. This can be compared to the use of *living labs* approaches [46] in disciplines such as human-computer interaction, interaction design, and computer-supported collaborative work where emphasis on context, work processes, and practices is increasingly considered important [47, 48]. To be able to create immersive prehospital scenarios, it is important that a simulation laboratory offers the means for close tailoring between required competence, methods and equipment/facilities, and the research problems and context presented. [37] Hence, it needs to provide both the width and depth of real prehospital scenarios. This can be attained through serious game-inspired techniques and methods, including sound and image projections, role-playing, and inclusion of all phases in an ambulance mission, along with data collection equipment and people with experience and competence in simulation development and design. An advanced prehospital simulation-based laboratory presents great promises to increase the scientific knowledge of prehospital care.
